# Sex dimorphic associations of Prader–Willi imprinted gene expressions in umbilical cord with prenatal and postnatal growth in healthy infants

**DOI:** 10.1007/s12519-024-00865-4

**Published:** 2025-01-22

**Authors:** Berta Mas-Parés, Gemma Carreras-Badosa, Ariadna Gómez-Vilarrubla, Antonio De Arriba-Muñoz, Olivia Lafalla-Bernard, Anna Prats-Puig, Francis De Zegher, Lourdes Ibañez, Andrea M. Haqq, Judit Bassols, Abel Lopez-Bermejo

**Affiliations:** 1 Pediatric Endocrinology, Girona Biomedical Research Institute, Hospital Dr. JosepTrueta, 17007 Girona, Spain; 2 Materno-Fetal Research, Girona Biomedical Research Institute, Parc Hospitalari Martí I Julià, Edifici M2, Salt, 17190 Girona, Spain; 3 Pediatric Endocrinology Unit, Miguel Servet Hospital, Saragossa, Spain; 4 San Jorge Hospital, Huesca, Spain; 5 University School of Health and Sport (EUSES), University of Girona, Girona, Spain; 6 Department of Development & Regeneration, University of Leuven, Louvain, Belgium; 7 Endocrinology, Sant Joan de Déu Children’s Hospital Pediatric Institute, University of Barcelona, Barcelona, Spain; 8 Pediatric Endocrinology and Metabolism, University of Alberta, Alberta, Canada; 9 Pediatrics, Dr. Josep Trueta Hospital, Girona, Spain; 10 Departament de Ciències Mèdiques, Universitat de Girona, Girona, Spain

**Keywords:** Gene expression, Imprinting, Postnatal growth, Prader–Willi syndrome, Sexual dimorphism

## Abstract

**Background:**

The impact of Prader–Willi syndrome (PWS) domain gene expression on the growth of healthy children is not well understood. This study investigated associations between PWS domain gene expression in umbilical cord tissue and prenatal and postnatal growth, considering potential sex differences.

**Methods:**

Relative gene expression of paternally expressed *MAGEL2*, *NDN*, and *SNURF-SNRPN*, and the small nucleolar RNAs *SNORD116* and *SNORD115* were determined by real-time quantitative polymerase chain reaction in umbilical cord tissue from 122 healthy newborns (59 girls and 63 boys). Gene expression levels were correlated with auxological measures at birth, infancy, and childhood (ages 2, 4, and 6 years).

**Results:**

*MAGEL2*, *NDN*, *SNORD116*, and *SNORD115* expression in the umbilical cord was negatively associated with birth weight, length, and placental weight (*P* < 0.001). Postnatally, these genes were positively associated with weight and length at 3 months (*P* < 0.001) and weight gain from birth to ages 1, 2, and 4 years (*P* < 0.01). Negative associations at birth were stronger in girls (*P* < 0.001), while positive associations during infancy and childhood were stronger in boys (*P* < 0.001). *MAGEL2*, *SNORD116*, and *SNORD115* expression predicted early-postnatal growth, explaining the higher growth rate in boys compared to girls and accounting for sex differences up to 1.5 kg in weight and 3 cm in height during infancy.

**Conclusions:**

Paternally expressed PWS domain gene expression in the umbilical cord was negatively associated with prenatal growth and positively with early-postnatal growth in healthy infants. This gene expression may predict early human postnatal growth and promote the well-known sex dimorphism in growth. These results can also help in understanding the etiology of PWS, which remains unclear.

**Graphical abstract:**

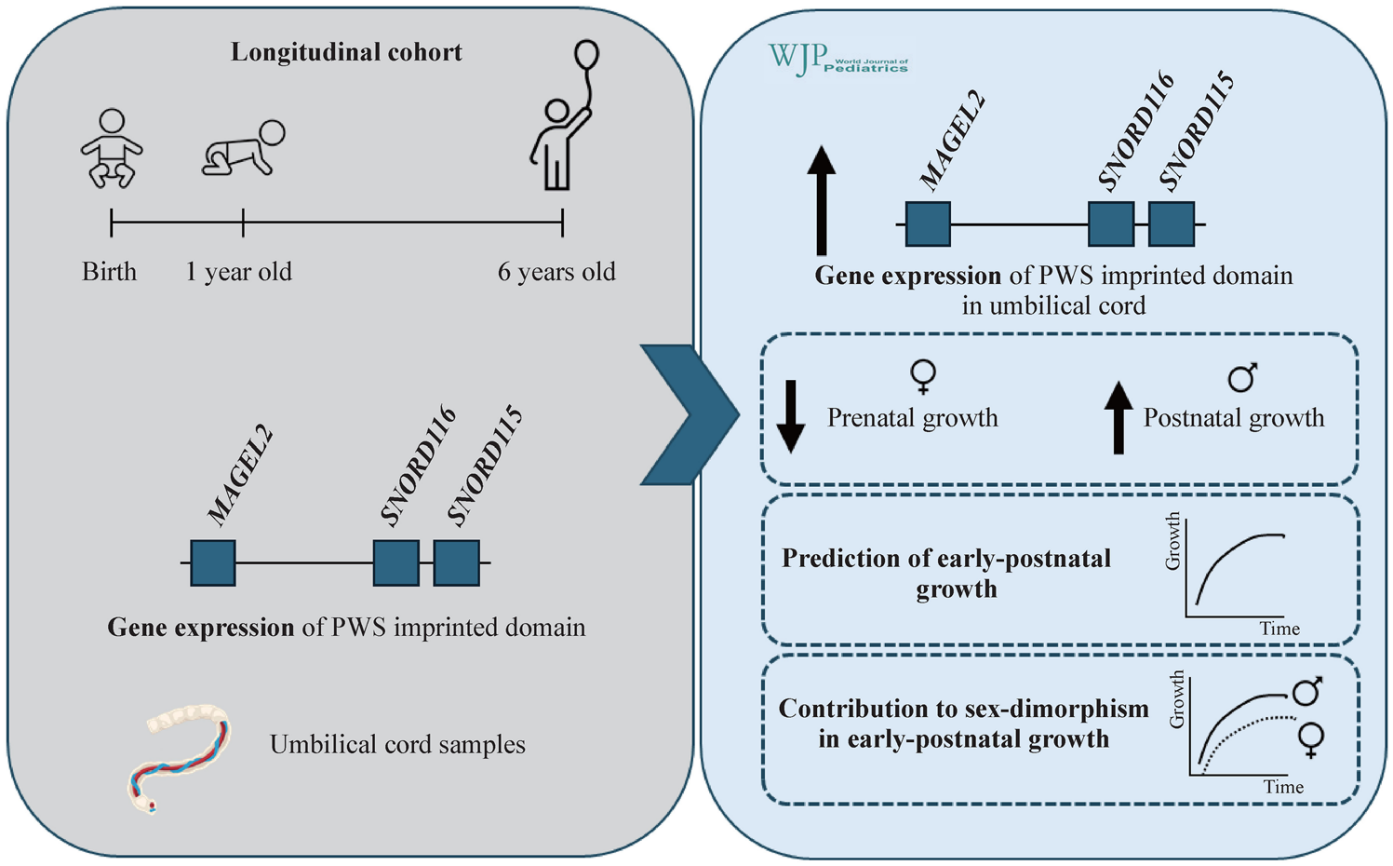

**Supplementary Information:**

The online version contains supplementary material available at 10.1007/s12519-024-00865-4.

## Introduction

Imprinted loci contain multiple genes expressed monoallelically in a parent-of-origin manner [1]. These loci are typically governed by an imprinting control region, which is epigenetically regulated and is subject to alterations affecting gene expression. The proper expression of imprinted genes is essential for normal prenatal development [2], and abnormalities can lead to various human pathologies, including Prader–Willi syndrome (PWS) [3].

PWS is an imprinted disorder marked by stunted growth in utero and infancy, followed by hyperphagia and excessive weight gain and adiposity beginning in early childhood [4, 5]. Indeed, the features related to PWS can be categorized into different nutritional phases, which correlate with infancy and childhood growth. The first phase is divided into two subphases: phase 1a (0–9 months), characterized by hypotonia and poor feeding, leading to failure to thrive, and phase 1b (9–25 months), where feeding normalizes and the infant begins to gain weight, though still below standard curves. Phase 2 generally begins around the age of two years and is also divided into two subphases: phase 2a (2–4 years), characterized by weight gain without notable changes in appetite or caloric intake, and phase 2b (4–8 years), involving continued weight gain, accompanied by a fixation on food. Phase 3 (around 8 years of age) is characterized by hyperphagia and an inability to achieve satiety [6–8].

PWS syndrome is caused by errors in imprinting maintenance on the chromosome 15q11-q13 region, leading to a loss of expression of the normally paternally expressed genes in the *SNURF-SNRPN/UBE3A* cluster [9, 10]. The absence of expression of one or more of these genes causes different phenotypes of PWS [5]. The *SNURF-SNRPN/UBE3A* cluster includes non-protein coding small nucleolar RNAs or snoRNAs (*SNORD116* and *SNORD115*), which have been related to prenatal growth and neurogenic development [11, 12], as well as paternally expressed protein-coding genes (*MAGEL2, NDN, and SNURF-SNRPN*). For instance, *MAGEL2* deficiency leads to behavioral abnormalities, fat infiltration in muscle tissue, disruptions in neurotransmitter signaling, and alterations in the volume of specific brain regions [13]. Despite extensive research implicating the *SNURF-SNRPN/UBE3A* cluster in prenatal growth, the role of these imprinted genes in postnatal development remains poorly understood.

Sex differences are well documented in various clinical outcomes, such as behavior [14], morphological traits, and disease risk [15]. Neonatal outcomes, such as birth weight and length, also demonstrate sex dimorphism in prenatal growth [15]. Since imprinted genes are related to prenatal growth, it is plausible that the regulation of imprinted genes may be influenced by sex. Previous literature supports this idea, as differences between males and females have been observed in the regulation of various imprinted genes, such as *IGF2*, *H19* [16], and *PEG3* [17, 18].

The umbilical cord plays a crucial role in transporting oxygen, nutrients, and metabolites from the mother to the fetus [19]. Due to its pleiotropic functions, it is a critical tissue in fetal development [19, 20] and can influence the start of extrauterine life [21].

Our hypothesis was that the expression of genes in the *SNURF-SNRPN/UBE3A* cluster in umbilical cord tissue would be associated with prenatal and postnatal growth in healthy infants. Therefore, this study aimed to investigate longitudinal associations between the relative gene expression of the *SNURF-SNRPN/UBE3A* cluster in the umbilical cord and parameters related to prenatal and postnatal growth from birth to school age in apparently healthy infants. As a secondary aim, we sought to investigate whether these associations were influenced by sex.

## Methods

### Study population and ethics

The study population included 122 apparently healthy pregnant Caucasian women delivering apparently healthy term infants. Participants were recruited from prenatal primary care in Northeastern Spain during their first trimester. Information on pregnancy, labor, and delivery characteristics was retrieved from standardized medical records. Women with major medical, surgical, or obstetrical complications, including multiple pregnancies, hypertension, gestational diabetes, or preeclampsia, and newborns with malformations or asphyxia were excluded. Other exclusion criteria included the use of assisted reproductive technology [22] and drug or alcohol abuse during pregnancy. The protocol was approved by the Institutional Review Board of Dr. Josep Trueta Hospital, and informed written consent was obtained from all parents.

### Anthropometric assessments

Maternal weight and height were assessed, and body mass index (BMI) was calculated as weight divided by height squared (kg/m^2^). Maternal weight at the beginning of gestation was used as a proxy for pre-pregnancy weight.

After delivery, infant weight and length were measured using a calibrated scale and measuring board. Gestational age- and sex-adjusted *Z* scores for birth weight and length were calculated using regional norms. Birth weight and length were used as proxies for prenatal growth. Infants included at birth, whose parents agreed to participate further in the study, were followed up during infancy (at 1, 2, 3, 4, 6, and 12 months; *n* = 122) and early childhood (at 2, 4, and 6 years of age; *n* = 106).

During infancy and at ages 2 and 4 years, weight and length/height measures were retrieved from the standard medical records. At 6 years of age, a follow-up visit was performed. Weight was measured on a calibrated scale wearing light clothes, and height was measured with a Harpenden stadiometer without shoes. BMI and age- and sex-adjusted *Z* scores were calculated as previously described, using regional norms [23].

### Umbilical cord sample collection and gene expression analysis

Immediately after delivery, the umbilical cord was clamped and cut, and a section of tissue (1–4 cm long) was stored and preserved in RNAlater at – 80 ºC until processing. For RNA isolation, a portion of umbilical cord tissue without vessels and containing Wharton’s jelly was used. The relative gene expression of the *SNURF-SNRPN/UBE3A* cluster was analyzed (Supplementary Fig. 1). Total RNA was isolated using the RNeasy mini kit (Qiagen) and retrotranscribed with the MultiScribe reverse transcriptase (ThermoFisher Scientific) according to the manufacturers’ protocol. The following TaqMan gene expression assays were used to amplify the cDNA: selected genes *MAGEL2* (Hs00255922_s1), *NDN* (Hs00267349_s1), *SNURF-SNRPN* (Hs01374551_m1), *SNORD116* (Hs03309544_s1), *SNORD115* (Hs04231709_gH), and the housekeeping gene *GAPDH* (Hs02786624_g1). Reactions were run on a LightCycler 480 real-time PCR system (Roche Diagnostics) using light cycler master mix (Roche Diagnostics). The cycling conditions were as follows: 10 minutes at 95 ºC, followed by 45 cycles of 2 seconds at 95 ºC and 30 seconds at 60 ºC, with a final step of 30 seconds at 40 ºC. Relative mRNA levels were calculated according to the 2^−ΔCt^ method.

### Statistics

Statistical analyses were conducted using the SPSS 22.0 package (IBM Inc.). Non-normally distributed data were log-transformed to improve symmetry. Differences between the subgroups in quantitative clinical variables and relative gene expression were examined using the Student’s *t* test. The associations between relative gene expression in umbilical cord tissue and the children’s anthropometric variables were analyzed using Pearson correlation, followed by multiple regression analysis using the enter method to adjust for possible confounding variables. Logistic regression models were used to predict postnatal growth variables based on umbilical cord gene expression. Infants were divided into two groups based on gene expression values: those above the 50th percentile and those below it. R version 4.3.0 and the lme4-package were also used to run mixed models for repeated measures in order to examine differences in growth patterns between boys and girls over time. The significance level was set at *P* < 0.05.

## Results

We studied a total of 122 infants: 59 girls and 63 boys. Comparison between sexes revealed differences in length at birth, weight, and length during infancy, and weight, height, and BMI at 6 years of age (Supplementary Table 1), with boys being larger. However, the relative gene expression of *MAGEL2*, *NDN*, *SNURF-SNRPN*, *SNORD115*, and *SNORD116* were similar in both girls and boys (Table 1).

**Table 1:** Gene expression levels of the *SNURF/SNRPN* imprinted cluster domain in the whole cohort and in girls versus boys

Umbilical cord relative gene expression	All subjects (*n* = 122)	Girls (*n* = 59)	Boys (*n* = 63)	*P*
*MAGEL2* (2^−ACT^)	− 1.84 ± 0.72	− 0.18 ± 0.70	− 0.18 ± 0.75	NS
*NDN* (2^−ACT^)	− 1.10 ± 0.34	− 1.11 ± 0.36	− 1.10 ± 0.32	NS
*SNRPN* (2^−ACT^)	− 1.72 ± 0.30	− 1.77 ± 0.34	− 1.68 ± 0.24	NS
*SNORD116* (2^−ACT^)	− 0.90 ± 0.52	− 0.88 ± 0.54	− 0.93 ± 0.50	NS
*SNORD115* (2^−ACT^)	0.09 ± − 0.02	0.12 ± 0.54	0.05 ± 0.56	NS

Data are presented as mean ± standard deviation values. Data were log-transformed to improve symmetry. Student’s *t* test was performed for comparison. *NS* not significant

### Associations of umbilical cord gene expression of the *SNURF-SNRPN/UBE3A* with prenatal and postnatal growth from birth to 6 years of age

The relative gene expression of the *SNURF-SNRPN/UBE3A* imprinted cluster in the umbilical cord correlated with several auxological measures from birth to 6 years. *MAGEL2* relative expression was independently and negatively associated with birth weight and length, while positively associated with weight and length at 3 months, weight catch-up at 1, 2, 4, and 6 years, and changes in BMI-to-birth weight standard deviation score (SDS) change at those ages (Table 2). *NDN*, *SNORD116*, *and SNORD115* relative expressions showed similar patterns, with independent and negative associations to birth weight, birth length and placental weight, and positive associations to weight and length at 3 months of age, and to changes in BMI-to-birth weight SDS at 6 years of age (Table 2).

**Table 2:** Correlations between the relative gene expression of *MAGEL2, NDN, SNURF-SNRPN, SNORD116* and *SNORD115* in the umbilical cord and the studied auxological variables in all the infants of the cohort and in girls versus boys

Variables	All subjects					Girls					Boys				
	*MAGEL2*	*NDN*	*SNURF-SNRPN*	*SNORD116*	*SNORD115*	*MAGEL2*	*NDN*	*SNURF-SNRPN*	*SNORD116*	*SNORD115*	*MAGEL2*	*NDN*	*SNURF-SNRPN*	*SNORD116*	*SNORD115*
At birth	*N* = 122					*n* = 59					*n* = 63				
Birth weight (g)	− 0.210^*^	− 0.245^*^	0.226^*^	**− 0.325**^**†**^	**− 0.337**^**†**^	− 0.269^*^	− 0.306^*^	**0.347**^**†**^	**− 0.439**^**†**^	**− 0.337**^**†**^	− 0.166	− 0.188	0.004	− 0.194	**− 0.334**^**†**^
Birth weight SDS (*Z* score)	− 0.313^*^	**− 0.339**^**†**^	0.201^*^	**− 0.393**^**†**^	**− 0.411**^**†**^	− 0.388^**†**^	− 0.365^**†**^	**0.393**^**†**^	**− 0.469**^**†**^	**− 0.415**^**†**^	− 0.241	− 0.310^*^	− 0.069	− 0.275^*^	**− 0.407**^**†**^
Birth length (cm)	− 0.221^*^	− 0.209^*^	0.156	**− 0.372**^**†**^	**− 0.320**^**†**^	**− 0.358**^**†**^	− 0.305^*^	0.250	**− 0.530**^**†**^	**− 0.430**^**†**^	− 0.098	− 0.108	− 0.052	− 0.189	− 0.207
Birth length SDS (*Z* score)	**− 0.306**^**†**^	**− 0.285**^**†**^	0.095	**− 0.432**^**†**^	**− 0.369**^**†**^	**− 0.459**^**†**^	− 0.351^**†**^	0.259	**− 0.587**^**†**^	**− 0.505**^**†**^	− 0.158	− 0.209	− 0.151	− 0.252^*^	− 0.242
Placental weight (g)	− 0.184	− 0.234^*^	0.022	**− 0.384**^**†**^	**− 0.337**^**†**^	**− 0.401**^**†**^	− 0.314^*^	0.045	**− 0.578**^**†**^	**− 0.538**^**†**^	0.053	− 0.130	0.001	− 0.127	− 0.099
At 1st y of infancy	*N* = 122					*n* = 59					*n* = 63				
1st mon weight (g)	0.027	− 0.047	0.126	− 0.191^*^	− 0.257^†^	− 0.047	− 0.120	0.191	− 0.328^*^	− 0.332^*^	0.072	− 0.008	− 0.050	− 0.053	− 0.186
1st mon length (cm)	− 0.032	− 0.064	0.145	− 0.185	− 0.229^*^	− 0.164	− 0.110	0.254	− 0.386^†^	**− 0.396**^**†**^	0.062	− 0.058	0.023	− 0.009	− 0.071
2nd mon weight (g)	0.090	− 0.002	0.200^*^	− 0.076	− 0.065	− 0.235	− 0.205	0.220	− 0.396^†^	− 0.423^†^	**0.383**^**†**^	0.220	0.069	0.262	0.299^*^
2nd mon length (cm)	0.009	− 0.042	0.178	− 0.109	− 0.067	− 0.235	− 0.197	0.200	− 0.425^†^	− 0.355^*^	0.241	0.145	0.070	0.248	0.240^*^
3rd mon weight (g)	**0.362**^**†**^	**0.287**^**†**^	0.059	0.234^*^	0.261^*^	0.095	0.087	− 0.012	− 0.060	− 0.118	**0.464**^**†**^	**0.367**^**†**^	0.076	0.332^*^	**0.441**^**†**^
3rd mon length (cm)	**0.357**^**†**^	**0.370**^**†**^	0.129	0.235^*^	0.243^*^	0.035	0.145	0.033	− 0.159	− 0.160	**0.513**^**†**^	**0.503**^**†**^	0.200	**0.427**^**†**^	**0.463**^**†**^
4th mon weight (g)	0.143	0.112	0.120	− 0.009	− 0.009	− 0.292^*^	− 0.202	0.125	− 0.395^†^	**− 0.442**^**†**^	**0.467**^**†**^	**0.374**^**†**^	0.062	0.320^*^	0.364^*^
4th mon length (cm)	0.150	0.079	0.163	− 0.026	0.019	− 0.263	− 0.207	0.190	**− 0.444**^**†**^	**− 0.409**^**†**^	**0.528**^**†**^	**0.372**^**†**^	0.072	**0.405**^**†**^	**0.462**^**†**^
6th mon weight (g)	0.176	0.125	0.044	0.004	0.039	− 0.135	− 0.065	0.044	− 0.263	− 0.335^*^	**0.438**^**†**^	0.291^*^	−0.065	0.258	**0.377**^**†**^
6th mon length (cm)	0.121	0.065	0.171	− 0.032	0.006	− 0.156	− 0.139	0.179	− 0.337^*^	− 0.300^*^	**0.381**^**†**^	0.275^*^	0.086	0.289^*^	0.317^*^
12th mon weight (g)	0.143	0.026	0.068	− 0.048	− 0.005	− 0.112	− 0.101	− 0.046	− 0.252	− 0.338^*^	**0.340**^**†**^	0.115	0.100	0.138	0.280^*^
12th mon weight SDS (*Z* score)	0.132	0.016	0.039	− 0.054	− 0.008	− 0.113	− 0.093	− 0.046	− 0.254	− 0.340^*^	0.334^*^	0.116	0.105	0.133	0.280^*^
12th mon weight SDS–birth weight SDS (catch−up) (*Z* score)	**0.331**^**†**^	0.241^*^	− 0.078	0.238^*^	0.286^†^	0.187	0.162	− 0.297^*^	0.163	− 0.025	**0.434**^**†**^	0.306^*^	0.126	0.304^*^	**0.515**^**†**^
12th mon BMI SDS–birth weight SDS (*Z* score)	**0.277**^**†**^	0.254^†^	− 0.127	0.241^†^	0.278^†^	0.237	0.240	− 0.324^*^	0.295^*^	0.098	0.304^*^	0.265^*^	0.067	0.199	0.408^†^
At age 2 y	*N* = 106					*n* = 52					*n* = 54				
Weight (g)	0.094	0.000	0.020	− 0.069	− 0.027	− 0.159	− 0.130	− 0.109	− 0.276^*^	− 0.304^*^	0.286^*^	0.094	0.043	0.132	0.222
Weight SDS (*Z* score)	0.093	− 0.006	− 0.003	− 0.068	− 0.025	− 0.159	− 0.130	− 0.109	− 0.276^*^	− 0.304^*^	0.286^*^	0.094	0.043	0.132	0.222
Weight SDS–birth weight SDS (catch−up) (*Z* score)	**0.329**^**†**^	0.277^†^	− 0.128	0.251^†^	0.301^†^	0.298	0.266	**− 0.421**^**†**^	0.252	0.128	**0.370**^**†**^	0.288^*^	0.094	0.266^*^	0.435^*^
BMI SDS–birth weight SDS (*Z* score)	**0.332**^**†**^	0.309^†^	− 0.094	0.274^†^	0.314^†^	0.381^†^	0.362^†^	− 0.289^*^	0.406^†^	0.288^*^	0.293^*^	0.257^*^	0.088	0.162	0.345^†^
At age 4 y	*N* = 106					*n* = 52					*n* = 54				
Weight (g)	0.188	0.083	− 0.031	0.012	0.014	0.033	0.011	− 0.067	− 0.086	− 0.200	0.293^*^	0.122	− 0.077	0.090	0.177
Weight SDS (*Z* score)	0.170	0.068	− 0.058	0.003	0.000	0.033	0.011	− 0.067	− 0.086	− 0.200	0.293^*^	0.122	− 0.077	0.090	0.177
Weight SDS–birth weight SDS (catch−up) (*Z* score)	**0.396**^**†**^	0.336^†^	− 0.199	0.360^†^	0.335^†^	0.363^*^	0.343^*^	− 0.386^†^	0.399^*^	0.140	**0.415**^**†**^	0.324^*^	− 0.008	0.326^*^	**0.466**^**†**^
BMI SDS–birth weight SDS (*Z* score)	**0.366**^**†**^	0.350^†^	− 0.235^*^	0.346^†^	0.320^†^	**0.383**^**†**^	0.378^†^	− 0.384^†^	0.454^†^	0.209	0.365^†^	0.331^*^	− 0.040	0.255	0.403^†^
At age 6 y	*N* = 106					*n* = 52					*n* = 54				
Weight SDS–birth weight SDS (catch−up) (*Z* score)	**0.267**^**†**^	**0.252**^**†**^	− 0.169	0.221^*^	**0.296**^**†**^	0.263	0.290^*^	− 0.241	0.334^*^	0.277^*^	0.266^*^	0.200	− 0.139	0.125	0.321^*^
BMI SDS–birth weight SDS (Z score)	**0.272**^**†**^	**0.266**^**†**^	− 0.137	**0.264**^**†**^	**0.341**^**†**^	0.307^*^	0.319^*^	− 0.168	**0.412**^**†**^	**0.385**^**†**^	0.240	0.201	− 0.130	0.125	0.305^*^

Multivariate analysis at birth (confounding variables: pre-pregnancy weight, mother’s height, sex of the infant and GA); multivariate analysis at age 1, 2, 4, and 6 years (confounding variables: pre-pregnancy weight, birth weight and GA, and age at follow-up). Significant results after correcting for confounding variables in multivariate analysis are shown in bold. Pearson *r* coefficients from bivariate correlation analysis are shown (^*^P < 0.05, ^†^*P* < 0.001). *SDS* standard deviation score, *BMI* body mass index, *GA* gestational age

### Sex dimorphic associations of umbilical cord gene expression of the *SNURF-SNRPN*/*UBE3A* cluster with prenatal and postnatal growth

The negative associations between umbilical cord gene expression of the *SNURF-SNRPN/UBE3A* cluster and growth parameters at birth were more pronounced in girls (*P* < 0.001). Conversely, the positive associations during infancy and childhood were stronger in boys (*P* < 0.001).

**Fig. 1:**
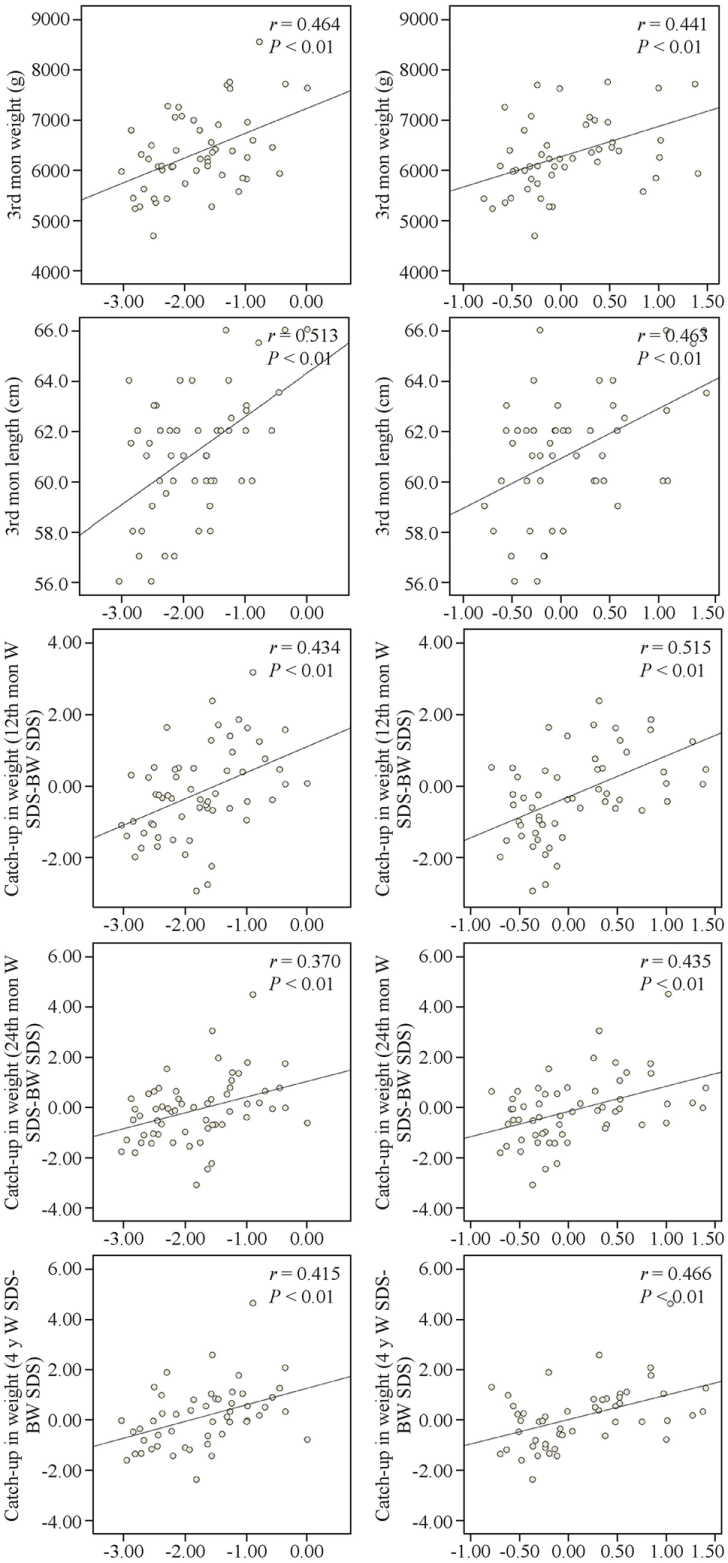
Correlations between the relative gene expression of *MAGEL2* and *SNORD115* in the umbilical cord and auxological variables during early childhood (postnatal growth) in boys. Pearson correlation coefficient (*r*) and *P* values are shown. *W* weight, *BW* birth weight, *SDS* standard deviation score

Girls exhibited independent and negative associations between the relative gene expression of *MAGEL2*, *SNORD116*, and *SNORD115* with birth weight, birth length, and placental weight (Supplementary Fig. 2). Later in life, girls showed independent but positive associations between the relative gene expression of *MAGEL2*, *NDN*, *SNORD116*, and *SNORD115* with BMI-to-birth weight SDS change at 4 and 6 years of age (Table 2). Boys showed independent and negative associations between the relative gene expression of *NDN*, *SNORD116*, and *SNORD115* and birth weight, but later in life independent and positive associations emerged between the relative gene expression of *MAGEL2* and *SNORD115* with weight and length from 2 months of age until 1 year of age, weight and length/height at 2 and 4 years of age, and weight catch-up at 1, 2, 4, and 6 years of age (Table 2 and Fig. 1).

### Prediction of early-postnatal growth by umbilical cord gene expression of the *SNURF-SNRPN*/*UBE3A* cluster

**Fig. 2:**
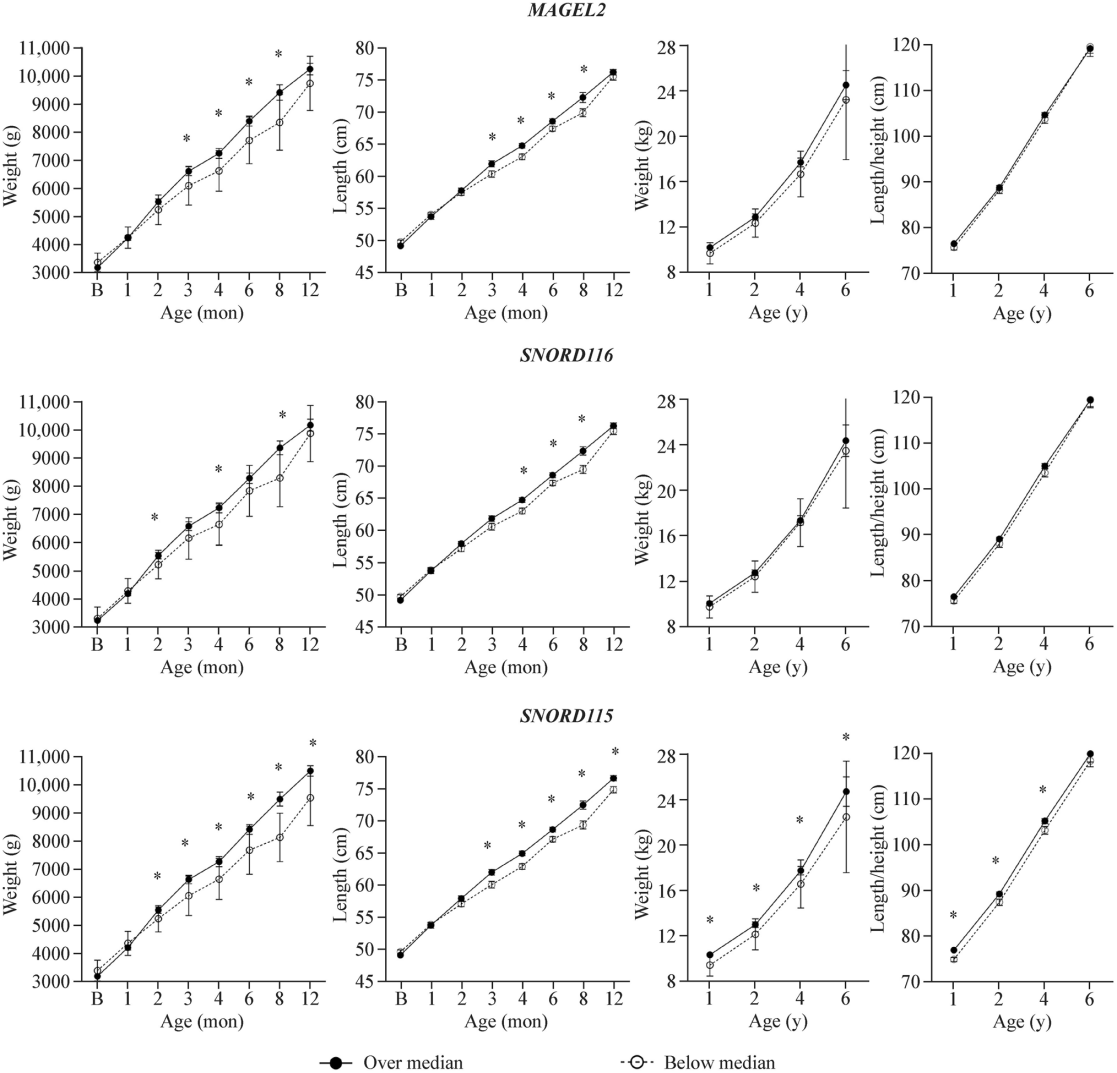
Weight and length/height curves in boys during infancy and from the first year until 6 years, according to a 50th centile (median) cutoff value of cord gene expression. ^*^P < 0.05

We evaluated whether umbilical cord gene expression levels could predict postnatal growth. Figure 2 shows the divergence in postnatal growth curves for boys during infancy, as well as from 1 to 6 years of age, based on the expression levels of the *SNURF-SNRPN/UBE3A* imprinted cluster in the umbilical cord. Boys with above-median expression values (50th centile) of *MAGEL2*, *SNORD116*, and *SNORD115* expression exhibited greater postnatal growth than those with gene expression levels below the median (Fig. 2). In boys, the odds ratio for higher weight at 1 year of age was 6.33 [*P* = 0.002, 95% confidence interval (CI) = 1.97–20.34], at 2 years of age was 5.83 (*P* = 0.002, 95% CI = 1.88–18.09), at 4 years of age was 3.38 (*P* = 0.039, 95% CI = 1.06–10.71), and at 6 years of age was 4.13 (*P* = 0.018, 95% CI = 1.27–13.39) when expressing higher levels of cord *SNORD115* (values above the median). The predictive ability of cord *MAGEL2*, *SNORD116*, and *SNORD115* expression was less apparent in girls (Supplementary Fig. 3).

### Umbilical cord gene expression of the *SNURF-SNRPN/UBE3A* cluster explains sexual dimorphism in early human postnatal growth

**Fig. 3:**
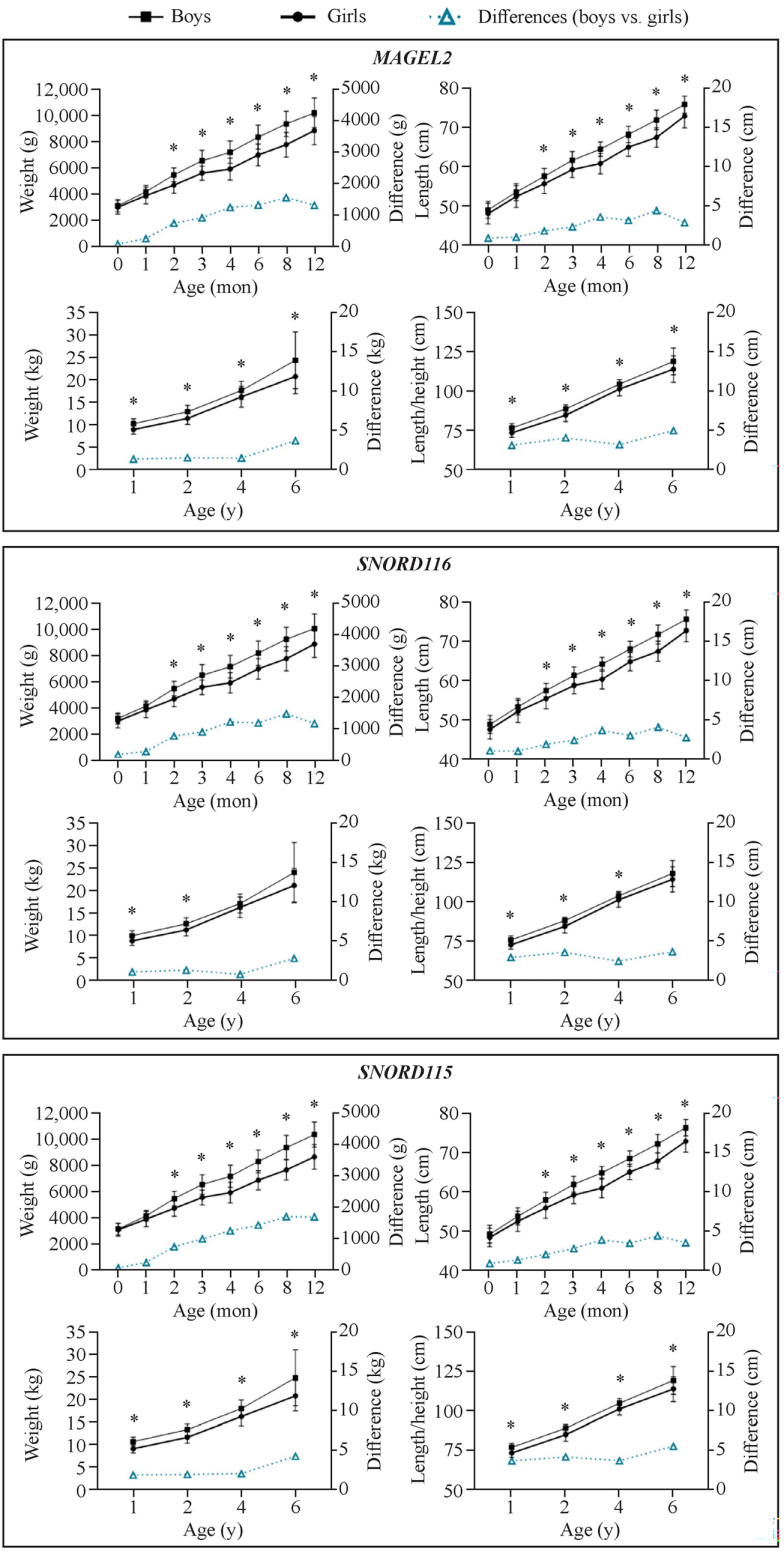
Differences between boys and girls in postnatal weight and length/height in infants with higher cord gene expression (those with expression levels above the 50th centile). ^*^P < 0.05

We investigated whether umbilical cord gene expression levels could explain differences in postnatal growth between boys and girls. In infants with expression levels of *MAGEL2*, *SNORD116*, and *SNORD115* above the median (Fig. 3), but not in those below the median (Supplementary Fig. 4), the postnatal growth curves of boys and girls diverged significantly (all *P* < 0.05), with boys exhibiting higher early-postnatal growth.

These observations were corroborated by the results of mixed models for repeated measures in the higher gene expression group, where the interaction between sex and time was statistically significant for all three genes during the first year of life (*P* < 0.001). Boys exhibited more pronounced growth than girls in both weight and length over time (Supplementary Tables 2–4). Specifically, boys with higher *MAGEL2* expression showed an average weight difference of up to 1 kg starting at 4 months of age compared to girls, along with a 2 cm greater length (Supplementary Table 2). Similar trends and effect sizes were observed for *SNORD116* (Supplementary Table 3). Finally, boys with higher levels of *SNORD115* presented a weight difference of over 1.5 kg starting at 6 months of age compared to girls, along with approximately 3 cm greater length (Supplementary Table 4).

In addition, multivariate mixed models for repeated measures, which included all studied infants, gene expression, as a dichotomous variable, was not directly associated with growth parameters. However, significant interactions between gene expression and sex persisted in their association with growth parameters in these models (Supplementary Table 5).

## Discussion

In the present study, we observed negative associations between the relative umbilical cord gene expression of the *SNURF-SNRPN/UBE3A* cluster and several parameters at birth, which were more pronounced in girls. Postnatally, we observed positive associations between the relative expression of this cluster and several postnatal growth parameters, with the associations being more significant in boys. Overall, these results suggest that gene expression within the PWS imprinted domain exhibits a sexual dimorphic association with both prenatal and postnatal growth in apparently healthy children. The differential associations observed at distinct time points (at birth and postnatally) are consistent with the nutritional phases seen in PWS (as described in the Introduction) [6, 7, 24] and may reflect differential effects of PWS genes on nutrition and growth, depending on the developmental stage and sex of the individual.

It is widely accepted that paternally expressed imprinted genes enhance growth, particularly fetal growth [2], and thus, it is expected that the paternally expressed *SNURF-SNRPN/UBE3A* cluster influences prenatal growth. Furthermore, fetuses have the ability to adapt to their external environment and make anticipatory responses due to their developmental plasticity [25]. In our study of healthy infants, we observed that smaller infants at birth had increased expression of growth-related genes. This may indicate a compensatory mechanism to improve prenatal growth, whereby a paternal line drives maternal resources to maximize fetal nutrition and optimize growth in utero and in infancy prior to weaning [26]. This proposed mechanism of compensation provides the fetus with a survival advantage [27], since fetal growth is a crucial factor that determines health and disease risk later in childhood and adulthood [28].

*MAGEL2* is known to be highly expressed in the hypothalamus [29] and plays a role in the regulation of whole-body energy metabolism [30]. It is closely associated with circadian rhythms [5], changes in leptin sensitivity [5, 31, 32] and the regulation of muscle mass, among others [32]. In this study, we found that *MAGEL2* expression in boys positively correlated with infant growth from the first months of life to 6 years, highlighting a previously unknown relationship between *MAGEL2* expression and growth in apparently healthy infants. *SNORD116* is thought to have a crucial role in PWS [5], since different alterations in this gene, including microdeletions, have been related to PWS-like phenotypes [33]. Furthermore, Zhang et al. [34] showed differences in *SNORD116* expression at birth compared to postnatal stages such as weaning and adulthood, indicating that its expression may be regulated throughout development. Our results also show that *SNORD116* correlations at birth and in the following years move in opposite directions, supporting the idea that this gene could have an important role in the PWS growth phenotype and is developmentally regulated. Finally, *SNORD115* has been also linked to the obese phenotype observed in PWS, because it is involved in the regulation of alternative splicing of the serotonin receptor 2C [35], which is known to influence appetite regulation and energy balance [36–38]. Stronger associations in infancy were found in the present study, where the expression *SNORD115* showed a positive correlation with infant growth at various time points.

We found that the gene expression of the *SNURF-SNRPN/UBE3A* cluster was positively associated with several parameters of postnatal growth, both during the first year of life and until 6 years of age. This suggests that the expression of the studied genes may influence postnatal growth beyond birth and into early childhood. Notably, infants with PWS typically develop obesity later in life, suggesting a strong postnatal developmental component to PWS-associated obesity. Some authors have hypothesized that the delayed onset of weight gain in early infancy observed in PWS may be attributed to the loss of *MAGEL2*, where its expression may progressively favor the development of leptin insensitivity postnatally, thereby contributing to the obesity phenotype associated with PWS [29, 32], as demonstrated in animal models [39]. Others have reported that abnormalities in oxytocin secretion contribute to the development of obesity in PWS patients, as oxytocin regulates food intake, energy expenditure, thermogenesis, and muscle tone and mass at different developmental stages in these individuals [40].

We identified sexually dimorphic differences in the associations of the *SNURF-SNRPN/UBE3A* domain with prenatal and postnatal growth. In girls, the associations at birth were found to be stronger, while in boys, the associations with postnatal growth were more evident. Although many imprinted genes are differentially expressed according to sex [14], we did not find any sex differences in the expression within our population. Our results support the notion that the phenotype of PWS is mainly acquired via hypothalamic dysfunction, including alterations in growth hormone (GH) secretion, abnormal body composition [41], and impaired thermoregulation [42]. The hypothalamus is a region of the brain that is highly dimorphic according to sex, which could partially explain the sexually dimorphic associations observed in our study [34]. In addition, the secretion of GH in adult rats has also been found to be sexually dimorphic, and both neonatal and adult steroid environments can influence the adult GH secretory pattern [43]. Fluctuations in sex hormone levels that lead to a different regulation of the hypothalamic–pituitary axis may potentially account for the differences observed between boys and girls during infancy, coinciding with the activation of the hypothalamic–pituitary–gonadal axis, a phenomenon known as minipuberty [44]. During this period, boys experience a significant increase in testosterone, which may impact their somatic development, while no similar effect has been observed in girls [45]. This testosterone surge could also influence the role of the studied genes in somatic growth. Sex-specific associations of imprinted genes with weight parameters at birth and postnatally have also been reported. In a study focused on methylation of imprinted genes in cord blood leukocytes rather than their expression, differences were found between boys and girls when examining associations with parameters of weight status, such as birth weight, weight for length at 1 year of age, and BMI at 3 years of age [46].

It was also observed that the relative expression of the studied genes in the umbilical cord was associated with postnatal growth curves during infancy and up to 4 years of age. Specifically, higher expression of *MAGEL2*, *SNORD116*, and *SNORD115* in boys was associated with greater postnatal growth. This indicates that the expression of these genes in the umbilical cord is not only associated with early-postnatal growth but can also help predict weight gain during the first years of life. This observation aligns with the theory postulated by Campbell [47], which states that paternal genes enhance postnatal growth by promoting appetite and suckling ability. Further, as mentioned previously, patients with PWS experience dysfunction of the hypothalamic–pituitary axis [48], which regulates the secretion of GH, a potent stimulator of growth [49]. Thus, it is not surprising that genes implicated in PWS affect postnatal growth, potentially acting as important growth regulators at the hypothalamic level.

Finally, we also found that the cord expression of *MAGEL2*, *SNORD116*, and *SNORD115* contributed to explaining the sexually dimorphic pattern observed in early-postnatal growth, with divergent postnatal growth curves between boys and girls being apparent in infants with gene expression levels above the median. However, in multivariate models that included all infants studied, gene expression, as a dichotomous variable, was not directly associated with growth parameters, likely because such associations were only evident the group with higher gene expression. Notwithstanding these findings, gene expression did show persistent significant interactions with sex in its association with growth parameters. These results indicate that gene expression in the PWS imprinted domain may promote the well-known sexual dimorphism in humans, where boys grow faster at an earlier age. We suggest that higher expression levels of these imprinted genes interact with the testosterone surge during minipuberty in boys, which may help explain, at least in part, the dimorphic pattern of early human postnatal growth. However, further research is required to validate these hypotheses.

This study is the first of its kind to establish an association between genes related to PWS and normal prenatal and postnatal growth in healthy children. Addressing this knowledge gap is crucial for understanding how growth is regulated, which can help prevent potential complications later in life. Furthermore, the umbilical cord, a transitory tissue that is easily accessible for sampling, can possess prognostic value, as indicated in this study by the prediction of postnatal growth trajectories based on cord gene expression [50].

It should be noted that one limitation of this study is that we did not assess either DNA methylation status or protein levels of the studied genes due to limited resources. Although DNA methylation plays a key regulatory role in imprinted genes, expression is downstream of DNA regulatory factors and therefore gene expression is potentially more functionally relevant. In addition, while birth weight was used as a proxy for prenatal growth, ultrasound data collected throughout pregnancy would provide a more accurate quantification of prenatal growth. In addition, given the small sample size of our study, further longitudinal studies are needed to corroborate our findings.

To summarize, we report that the expression levels of paternally expressed genes from the PWS domain in the umbilical cord were negatively associated with prenatal growth but positively with early-postnatal growth in apparently healthy infants (Supplementary Table 6). This suggests that these genes may provide an advantage during the perinatal period and early-postnatal life, first to the mother–fetus pair and then to the young infant. In boys, the expression of these genes in the umbilical cord could reflect early effectors of growth patterns and may help predict weight gain during the first years of postnatal life. Lastly, gene expression levels of *MAGEL2*, *SNORD116*, and *SNORD115* may contribute to the well-known sexual dimorphism in humans, whereby boys grow faster at an earlier age.

## Supplementary Information

Below is the link to the electronic supplementary material.

Supplementary file 1 (PDF 882 kb)

Supplementary file 2 (PDF 772 kb)

## Data Availability

The datasets generated during and/or analyzed during the current study are available from the corresponding author on reasonable request.
