# Supplementary material for: Sex dimorphic associations of Prader–Willi imprinted gene expressions in umbilical cord with prenatal and postnatal growth in healthy infants
Source: World J Pediatr. 2025 Jan 22;21(1):100–12. doi: 10.1007/s12519-024-00865-4 (PMC11813995; doi:10.1007/s12519-024-00865-4)
Supplement: Supplementary file 1 — (PDF 882 kb) [file 12519_2024_865_MOESM1_ESM.pdf]

Supplementary Table 1. Descriptive analysis of the studied variables in the whole cohort and in girls versus boys

Variables	All subjects	Girls	Boys	*P*
**At birth**	***N* = 122**	***n* = 59**	***n* = 63**	
Gestational age (wk)	39.6 ± 1.4	39.6 ± 1.5	39.6 ± 1.3	NS
Birth weight (g)	3211 ± 444	3133 ± 468	3283 ± 411	NS
Birth weight SDS (*Z* score)	-0.16 ± 0.99	-0.20 ± 1.06	-0.12 ± 0.94	NS
Birth length (cm)	49.1 ± 2.1	48.7 ± 2.3	49.5 ± 1.9	**0.040**
Birth length SDS (*Z* score)	-0.36 ± 1.17	-0.41 ± 1.24	-0.31 ± 1.10	NS
Placental weight (g)	583 ± 112	584 ± 122	582 ± 102	NS
**At 1st y of infancy**	***N* = 122**	***n* = 59**	***n* = 63**	
1st mon weight (g)	4112 ± 523	3965 ± 580	4252 ± 423	**0.004**
1st mon length (cm)	53.4 ± 2.2	52.8 ± 2.4	53.9 ± 1.9	**0.017**
2nd mon weight (g)	5143 ± 630	4866 ± 609	5393 ± 543	**< 0.001**
2nd mon length (cm)	57.0 ± 2.3	56.3 ± 2.4	57.6 ± 1.9	**0.006**
3rd mon weight (g)	6041 ± 783	5689 ± 625	6353 ± 781	**< 0.001**
3rd mon length (cm)	60.4 ± 2.4	59.6 ± 2.1	61.1 ± 2.5	**0.002**
4th mon weight (g)	6572 ± 906	6194 ± 822	6927 ± 842	**< 0.001**
4th mon length (cm)	62.8 ± 2.5	61.6 ± 2.5	63.8 ± 2.1	**< 0.001**
6th mon weight (g)	7605 ± 995	7158 ± 864	8029 ± 929	**< 0.001**
6th mon length (cm)	66.8 ± 2.5	65.6 ± 2.3	67.9 ± 2.1	**< 0.001**
12th mon weight (g)	9545 ± 1139	9084 ± 1027	9983 ± 1062	**< 0.001**
12th mon weight SDS (*Z* score)	-0.47 ± 0.95	-0.60 ± 0.95	-0.34 ± 0.95	NS
12th mon height (cm)	74.8 ± 2.8	73.78 ± 2.62	75.8 ± 2.7	**< 0.001**
12th mon length SDS (*Z* score)	-0.06 ± 1.03	-0.19 ± 1.06	0.05 ± 1.01	NS
12th mon BMI (kg/cm^2)	16.96 ± 1.35	16.64 ± 1.19	17.24 ± 1.43	**0.016**
12th mon BMI SDS (*Z* score)	-0.54 ± 0.86	-0.60 ± 0.74	-0.49 ± 0.96	NS
12th mon weight SDS–birth weight SDS (catch-up) (*Z* score)	-0.32 ± 1.18	-0.45 ± 1.10	-0.20 ± 1.25	NS
12th mon BMI SDS–birth weight SDS (*Z* score)	-0.41 ± 1.26	-0.46 ± 1.19	-0.36 ± 1.33	NS
**At age 2 y**	***N* = 106**	***n* = 52**	***n* = 54**	
Weight (kg)	12.1 ± 1.3	11.6 ± 1.2	12.6 ± 1.4	**< 0.001**
Weight SDS (*Z* score)	-0.37 ± 0.94	-0.58 ± 0.91	-0.18 ± 0.95	**0.024**
Length (cm)	87.2 ± 3.7	85.9 ± 3.8	88.4 ± 3.3	**< 0.001**
Length SDS (*Z* score)	-0.05 ± 1.27	-0.19 ± 1.37	0.08 ± 1.17	NS
BMI (kg/cm^2)	15.95 ± 1.18	15.76 ± 1.09	16.13 ± 1.24	NS
BMI SDS (*Z* score)	-0.44 ± 0.87	-0.60 ± 0.80	-0.30 ± 0.90	NS
Weight SDS–birth weight SDS (catch-up) (*Z* score)	-0.23 ± 1.17	-0.39 ± 1.02	-0.08 ± 1.28	NS
BMI SDS–birth weight SDS (*Z* score)	-0.30 ± 1.32	-0.40 ± 1.28	-0.20 ± 1.36	NS
**At age 4 y**	***N* = 106**	***n* = 52**	***n* = 54**	
Weight (kg)	16.8 ± 2.1	16.3 ± 2.1	17.2 ± 2.1	**0.047**
Weight SDS (*Z* score)	-0.07 ± 0.85	-0.11 ± 0.92	-0.04 ± 0.80	NS
Height (cm)	103.1 ± 3.9	101.9 ± 4.0	104.2 ± 3.5	**0.004**
Height SDS (*Z* score)	-0.08 ± 0.93	-0.27 ± 0.98	0.09 ± 0.86	**0.049**
BMI (kg/cm^2)	15.78 ± 1.43	15.71 ± 1.43	15.84 ± 1.45	NS
BMI SDS (*Z* score)	-0.14 ± 0.84	-0.07 ± 0.86	-0.22 ± 0.83	NS
Weight SDS–birth weight SDS (catch-up) (*Z* score)	0.05 ± 1.17	-0.01 ± 1.11	0.11 ± 1.22	NS

BMI SDS–birth weight SDS (*Z* score)	-0.02 ± 1.25	0.03 ± 1.24	-0.07 ± 1.27	NS
**At age 6 y**	***N* = 106**	***n* = 52**	***n* = 54**	
Age (y)	6.25 ± 0.89	6.15 ± 0.89	6.33 ± 0.88	NS
Weight (kg)	22.5 ± 5.0	21.2 ± 3.7	23.7 ± 5.7	**0.009**
Weight SDS (*Z* score)	-0.12 ± 1.01	-0.29 ± 0.87	0.04 ± 1.11	NS
Height (cm)	117.7 ± 8.2	116.1 ± 8.1	119.3 ± 8.0	**0.042**
Height SDS (*Z* score)	0.01 ± 1.21	-0.17 ± 1.26	0.19 ± 1.14	NS
BMI (kg/cm^2)	16.12 ± 2.00	15.70 ± 1.59	16.51 ± 2.26	**0.036**
BMI SDS (*Z* score)	-0.16 ± 0.87	-0.26 ± 0.76	-0.05 ± 0.95	NS
Weight SDS–birth weight SDS (catch-up) (*Z* score)	0.01 ± 1.20	-0.12 ± 1.15	0.14 ± 1.23	NS
BMI SDS–birth weight SDS	-0.03 ± 1.26	-0.10 ± 1.30	0.04 ± 1.24	NS

Data are shown as mean ± standard deviation values. Student's *t* test was performed for comparison. *SDS* standard deviation score, *BMI* body mass index, *NS* not significant

Supplementary Table 2. Repeated-measures linear mixed models for *MAGEL2* in infants with higher (above the median) *MAGEL2* gene expression (n = 63)

Weight (g)						
**Analysis of variance**						
Variables	Sum Sq	Mean Sq	NumDF	DenDF	*F* value	Pr (> *F*)
Time (mon)	1745743360	249391909	7	330.36	1.212	**< 2.2E-16**
Sex	6287019	6287019	1	60.13	30.547	**7.42E-07**
Time:sex	19777205	2825315	7	330.36	13.727	**1.33E-15**
**Mixed models for repeated measures**						
Variables	Coefficient	Std. error	*Df*	*t* value	Pr (> |*t*|)	
Sex (male)	134.0	195.9	114.5	0.68	0.49	
Time 1	187.4	170.0	332.6	1.10	0.27	
Time 2	575.4	171.9	332.7	3.35	**9.12E-04**	
Time 3	962.9	182.2	333.2	5.29	**2.26E-07**	
Time 4	1050.9	180.9	333.2	5.81	**1.46E-08**	
Time 6	1163.8	171.8	332.9	6.77	**5.71E-11**	
Time 8	1236.3	214.1	333.6	5.77	**1.76E-08**	
Time 12	1089.3	169.2	332.4	6.44	**4.28E-10**	
Length (cm)						
**Analysis of variance**						
Variables	Sum Sq	Mean Sq	NumDF	DenDF	*F* value	Pr (> *F*)
Time (mon)	27554.8	3936.4	7	319.83	2.230	**< 2.2E-16**
Sex	33.9	33.9	1	58.52	19.2047	**4.94E-05**
Time:sex	72.2	11	7	319.83	6.2479	**7.06E-07**
**Mixed models for repeated measures**						
Variables	Coefficient	Std. error	*Df*	*t* value	Pr (> |*t*|)	
Sex (male)	0.97	0.62	100.9	1.58	0.12	
Time 1	0.17	0.51	323.0	0.33	0.74	
Time 2	0.58	0.51	323.1	1.13	0.26	
Time 3	1.97	0.54	323.2	3.66	**2.97E-04**	
Time 4	2.05	0.53	323.2	3.87	**1.34E-04**	
Time 6	2.14	0.50	323.0	4.24	**2.94E-05**	
Time 8	2.34	0.63	323.1	3.72	**2.33E-04**	
Time 12	1.77	0.50	322.7	3.56	**4.32E-04**	

Analysis of variance from linear mixed models for repeated measures is shown in infants with higher (above the median) gene expression. Results indicate an interaction of sex with time in infant growth during the first year of life

Supplementary Table 3. Repeated-measures linear mixed models for *SNORD116* in infants with higher (above the median) *SNORD116* gene expression (n = 65)

Weight (g)						
**Analysis of variance**						
Variables	Sum Sq	Mean Sq	NumDF	DenDF	*F* value	Pr (> *F*)
Time (mon)	1756006867	250858124	7	330.3	1.299	**< 2.2E-16**
Sex	6293955	6293955	1	58.48	32.584	**4.02E-07**
Time:sex	18877477	2696782	7	330.3	13.961	**7.30E-16**
**Mixed models for repeated measures**						
Variables	Coefficient	Std. error	*Df*	*t* value	Pr (> |*t*|)	
Sex (male)	193.9	195.2	109.6	0.99	0.32	
Time 1	131.5	164.6	331.2	0.80	0.42	
Time 2	586.5	167.4	331.4	3.50	**5.24E-04**	
Time 3	968.4	175.8	331.9	5.51	**7.21E-08**	
Time 4	1068.5	174.9	331.9	6.11	**2.84E-09**	
Time 6	1075.3	166.4	331.5	6.46	**3.82E-10**	
Time 8	1191.9	202.3	332.7	5.89	**9.43E-09**	
Time 12	1006.9	163.9	331.3	6.14	**2.30E-09**	
Length (cm)						
**Analysis of variance**						
Variables	Sum Sq	Mean Sq	NumDF	DenDF	*F* value	Pr (> *F*)
Time (mon)	27554.8	3936.4	7	319.83	2.230	**< 2.2E-16**
Sex	33.9	33.9	1	58.52	19.2047	**4.94E-05**
Time:sex	72.2	11	7	319.83	6.2479	**7.06E-07**
**Mixed models for repeated measures**						
Variables	Coefficient	Std. error	*Df*	*t* value	Pr (> |*t*|)	
Sex (male)	1.26	0.61	104.7	2.04	**0.04**	
Time 1	0.22	0.53	320.8	0.40	0.69	
Time 2	0.64	0.53	320.9	1.21	0.23	
Time 3	1.98	0.55	320.9	3.61	**3.54E-04**	
Time 4	2.35	0.54	320.9	4.33	**2.02E-05**	
Time 6	2.05	0.52	320.6	3.96	**9.06E-05**	
Time 8	2.38	0.63	321.5	3.78	**1.84E-04**	
Time 12	1.92	0.51	320.5	3.77	**1.95E-04**	

Analysis of variance from linear mixed models for repeated measures is shown in infants with higher (above the median) gene expression. Results indicate an interaction of sex with time in infant growth during the first year of life

Supplementary Table 4. Repeated-measures linear mixed models for *SNORD115* in infants with higher (above the median) *SNORD115* gene expression (n = 65)

Weight (g)						
**Analysis of variance**						
Variables	Sum Sq	Mean Sq	NumDF	DenDF	*F* value	Pr (> *F*)
Time (mon)	1809447346	258492478	7	329.12	1.609.22	**< 2.2E-16**
Sex	9030600	9030600	1	58.28	56.219	**4.15E-10**
Time:sex	40835328	5833618	7	329.12	36.317	**< 2.2E-16**
**Mixed models for repeated measures**						
Variables	Coefficient	Std. error	*Df*	*t* value	Pr (> |*t*|)	
Sex (male)	48.3	178.7	108.8	0.27	0.79	
Time 1	231.5	149.7	329.9	1.55	0.12	
Time 2	804.1	153.9	330.3	5.23	**3.07E-07**	
Time 3	1233.6	157.5	330.5	7.83	**6.56E-14**	
Time 4	1371.7	162.3	330.9	8.45	**9.27E-16**	
Time 6	1573.0	153.1	330.4	10.27	**1.16E-21**	
Time 8	1837.8	187.1	331.6	9.82	**3.79E-20**	
Time 12	1686.6	149.5	330.0	11.28	**3.55E-25**	
Length (cm)						
**Analysis of variance**						
Variables	Sum Sq	Mean Sq	NumDF	DenDF	*F* value	Pr (> *F*)
Time (mon)	27674.1	3953.4	7	319.26	2.568	**< 2.2E-16**
Sex	43.6	43.6	1	57.28	28.334	**1.77E-06**
Time:sex	132.2	18.9	7	319.26	12.267	**6.69E-14**
**Mixed models for repeated measures**						
Variables	Coefficient	Std. error	*Df*	*t* value	Pr (> |*t*|)	
Sex (male)	0.80	0.59	97.5	1.35	0.18	
Time 1	0.38	0.48	320.9	0.79	0.43	
Time 2	1.16	0.48	321.2	2.39	**0.02**	
Time 3	2.46	0.49	321.2	4.99	**1.00E-06**	
Time 4	2.88	0.50	321.4	5.72	**2.38E-08**	
Time 6	2.61	0.48	320.9	5.50	**7.75E-08**	
Time 8	3.43	0.58	321.7	5.91	**8.66E-09**	
Time 12	2.71	0.46	320.8	5.84	**1.31E-08**	

Analysis of variance from linear mixed models for repeated measures are shown in infants with higher (above the median) gene expression. Results indicate an interaction of sex with time in infant growth during the first year of life

Supplementary Table 5. Repeated measures linear mixed models in the whole cohort

***MAGEL2* (*n* = 106)**						
Weight (g)						
Variables	Sum Sq	Mean Sq	NumDF	DenDF	*F* value	Pr (> *F*)
Time (mon)	3427906450	489700921	7	686.95	2338.3469	**2.2E-16**
Sex	6707316	6707316	1	120.55	32.0278	**1.05E-07**
Expression	105140	105140	1	119.41	0.502	4.80E-01
Time:sex	16220776	2317254	7	686.95	11.065	**2.69E-13**
Expression:sex	965620	965620	1	119.41	4.6109	**0.03379**
Length (cm)						
Variables	Sum Sq	Mean Sq	NumDF	DenDF	*F* value	Pr (> *F*)
Time (mon)	54304	7757.7	7	662.73	4,387	**2.2E-16**
Sex	40	40.5	1	117.47	22.8841	**5.04E-06**
Expression	0	0.2	1	116.45	0.1232	7.26E-01
Time:sex	66	9.4	7	662.73	5.3261	**6.07E-06**
Expression:sex	4	3.5	1	116.45	2.0042	0.1595
***SNORD116* (*n* = 106)**						
Weight (g)						
Variables	Sum Sq	Mean Sq	NumDF	DenDF	*F* value	Pr (> *F*)
Time (mon)	3412330380	487475769	7	681.13	2326.8102	**2.2E-16**
Sex	6617270	6617270	1	119.68	31.5854	**1.27E-07**
Expression	3394	3394	1	118.53	0.0162	8.99E-01
Time:sex	16594208	2370601	7	681.13	11.3153	**1.31E-13**
Expression:sex	1238023	1238023	1	118.53	5.9093	**0.01656**
Length (cm)						
Variables	Sum Sq	Mean Sq	NumDF	DenDF	*F* value	Pr (> *F*)
Time (mon)	53876	7696.6	7	657.05	4367.5168	**2.2E-16**
Sex	42	41.7	1	116.64	23.6835	**3.59E-06**
Expression	1	0.8	1	115.6	0.4769	4.91E-01
Time:sex	64	9.2	7	657.05	5.2114	**8.49E-06**
Expression:sex	10	9.5	1	115.6	5.695	**0.02194**
***SNORD115* (*n* = 106)**						
Weight (g)						
Variables	Sum Sq	Mean Sq	NumDF	DenDF	*F* value	Pr (> *F*)
Time (mon)	3332400021	476057146	7	670.42	2258.3827	**2.2E-16**
Sex	6635096	6635096	1	117.8	31.4764	**1.36E-07**
Expression	82493	82493	1	116.5	0.3913	5.33E-01
Time:sex	15404298	2200614	7	670.42	10.4396	**1.77E-12**
Expression:sex	3710462	3710462	1	116.5	17.6022	**5.34E-05**
Length (cm)						
Variables	Sum Sq	Mean Sq	NumDF	DenDF	*F* value	Pr (> *F*)
Time (mon)	52837	7548.1	7	646.03	4318.3471	**2.2E-16**
Sex	36	35.8	1	114.52	20.4576	**1.50E-05**
Expression	1	1	1	113.4	0.566	4.53E-01
Time:sex	67	9.5	7	646.03	5.4364	**4.46E-06**
Expression:sex	14	14.2	1	113.4	8.1482	**5.13E-03**

Analysis of variance from linear mixed models for repeated measures are shown for the whole cohort. Gene expression is used as a dichotomous variable (below and above the median). Results indicate an interaction of gene expression with sex in infant growth over time (during the first year of life)

Supplementary Table 6. Summary of the key correlations between the relative gene expression of *MAGEL2, NDN, SNURF-SNRPN, SNORD116* and *SNORD115* in the umbilical cord and the studied auxological variables in all the infants of the cohort (*N* = 122) and in girls versus boys

Variables	All subjects			Girls			Boys		
	MAGEL 2	*SNORD -116*	*SNORD-115*	*MAGEL 2*	*SNORD -116*	*SNORD -115*	*MAGEL 2*	*SNORD -116*	*SNORD -115*
**At birth**	***N* = 122**			***n* = 59**			***n* = 63**		
Birth weight-SDS (*Z* score)	**-0.313***	**-0.393†**	**-0.411†**	-0.388†	**-0.469†**	**-0.415†**	-0.241	**-0.275***	**-0.407†**
Birth length-SDS (*Z* score)	**-0.306†**	**-0.432†**	**-0.369†**	**-0.459†**	**-0.587†**	**-0.505†**	-0.158	-0.252*	-0.242
Placental weight (g)	-0.184	**-0.384†**	**-0.337†**	**-0.401†**	**-0.578†**	**-0.538†**	0.053	-0.127	-0.099
**At 1st y of infancy**	***N* = 122**			***n* = 59**			***n* = 63**		
1st mon weight (g)	0.027	-0.191*	-0.257†	-0.047	-0.328*	-0.332*	0.072	-0.053	-0.186
1st mon length (cm)	-0.032	-0.185	-0.229*	-0.164	-0.386†	**-0.396†**	0.062	-0.009	-0.071
3rd mon weight (g)	**0.362†**	**0.234***	**0.261***	0.095	-0.060	-0.118	**0.464†**	**0.332***	**0.441†**
3rd mon length (cm)	**0.357†**	**0.235***	**0.243***	0.035	-0.159	-0.160	**0.513†**	**0.427†**	**0.463†**
6th mon weight (g)	0.176	0.004	0.039	-0.135	-0.263	-0.335*	**0.438†**	0.258	**0.377†**
6th mon length (cm)	0.121	-0.032	0.006	-0.156	-0.337*	-0.300*	**0.381†**	**0.289***	**0.317***
12th mon weight (g)	0.143	-0.048	-0.005	-0.112	-0.252	-0.338*	**0.340†**	0.138	**0.280***
12th mon weight SDS (*Z* score)	0.132	-0.054	-0.008	-0.113	-0.254	-0.340*	**0.334***	0.133	**0.280***
12th mon weight SDS–birth weight SDS (catch-up) (*Z* score)	**0.331†**	0.238*	0.286†	0.187	0.163	-0.025	**0.434†**	0.304*	**0.515†**
12th mon BMI SDS–birth weight SDS (*Z* score)	**0.277†**	0.241†	0.278†	0.237	0.295*	0.098	0.304*	0.199	0.408†
**At age 2 y**	***N* = 106**			***n* = 52**			***n* = 54**		
Weight SDS (*Z* score)	0.093	-0.068	-0.025	-0.159	-0.276*	-0.304*	**0.286***	0.132	0.222
Weight SDS–birth weight SDS (catch-up) (*Z* score)	**0.329†**	0.251†	0.301†	0.298	0.252	0.128	**0.370†**	0.266*	**0.435***
BMI SDS–birth weight SDS (*Z* score)	**0.332†**	0.274†	0.314†	0.381†	0.406†	0.288*	0.293*	0.162	0.345†
**At age 4 y**	***N* = 106**			***n* = 52**			***n* = 54**		
Weight SDS (*Z* score)	0.170	0.003	0.000	0.033	-0.086	-0.200	**0.293***	0.090	0.177
Weight SDS–birth weight SDS (catch-up) (*Z* score)	**0.396†**	0.360†	0.335†	0.363*	0.399*	0.140	**0.415†**	0.326*	**0.466†**
BMI SDS–birth weight SDS (*Z* score)	**0.366†**	0.346†	0.320†	**0.383†**	0.454†	0.209	0.365†	0.255	0.403†
**At age 6 y**	***N* = 106**			***n* = 52**			***n* = 54**		
Weight SDS–birth weight SDS (catch-up) (*Z* score)	**0.267†**	**0.221***	**0.296†**	0.263	**0.334***	0.277*	**0.266***	0.125	**0.321***
BMI SDS–birth weight SDS (*Z* score)	**0.272†**	**0.264†**	**0.341†**	**0.307***	**0.412†**	**0.385†**	0.240	0.125	0.305*

Multivariate analysis at birth (confounding variables: pre-pregnancy weight, mother's height, sex of the infant and GA); multivariate analysis at age 1, 2 , 4 and 6 years (confounding variables: pre-pregnancy weight, birth weight and GA, and age at follow-up). Significant results after correcting for confounding variables in multivariate analysis are shown in bold. Pearson *r* coeficients from bivariate correlation analysis are shown (*$P < 0.05$, †$P < 0.001$). *SDS* standard deviation score, *BMI* body mass index, *GA* gestational age
