# Supplementary material for: Sex dimorphic associations of Prader–Willi imprinted gene expressions in umbilical cord with prenatal and postnatal growth in healthy infants
Source: World J Pediatr. 2025 Jan 22;21(1):100–12. doi: 10.1007/s12519-024-00865-4 (PMC11813995; doi:10.1007/s12519-024-00865-4)
Supplement: Supplementary file 2 — (PDF 772 kb) [file 12519_2024_865_MOESM2_ESM.pdf]

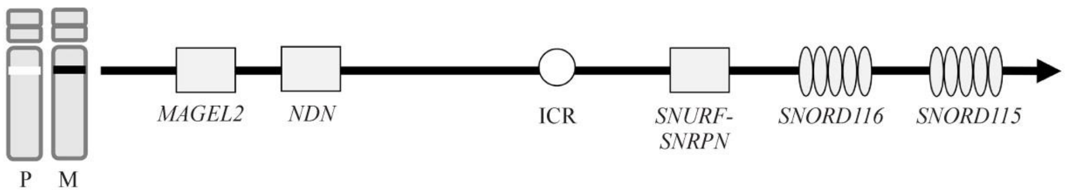

Supplementary Fig. 1 Schematic representation of human 15q11-13 region corresponding to the paternally expressed genes of the *SNURF-SNRPN/UBE3A* imprinted cluster (not to scale). Protein coding and SNORD expressed genes are marked as boxes and ovals, respectively. The imprinting control region (ICR) is schematically indicated by a circle. *P* paternal, *M* maternal

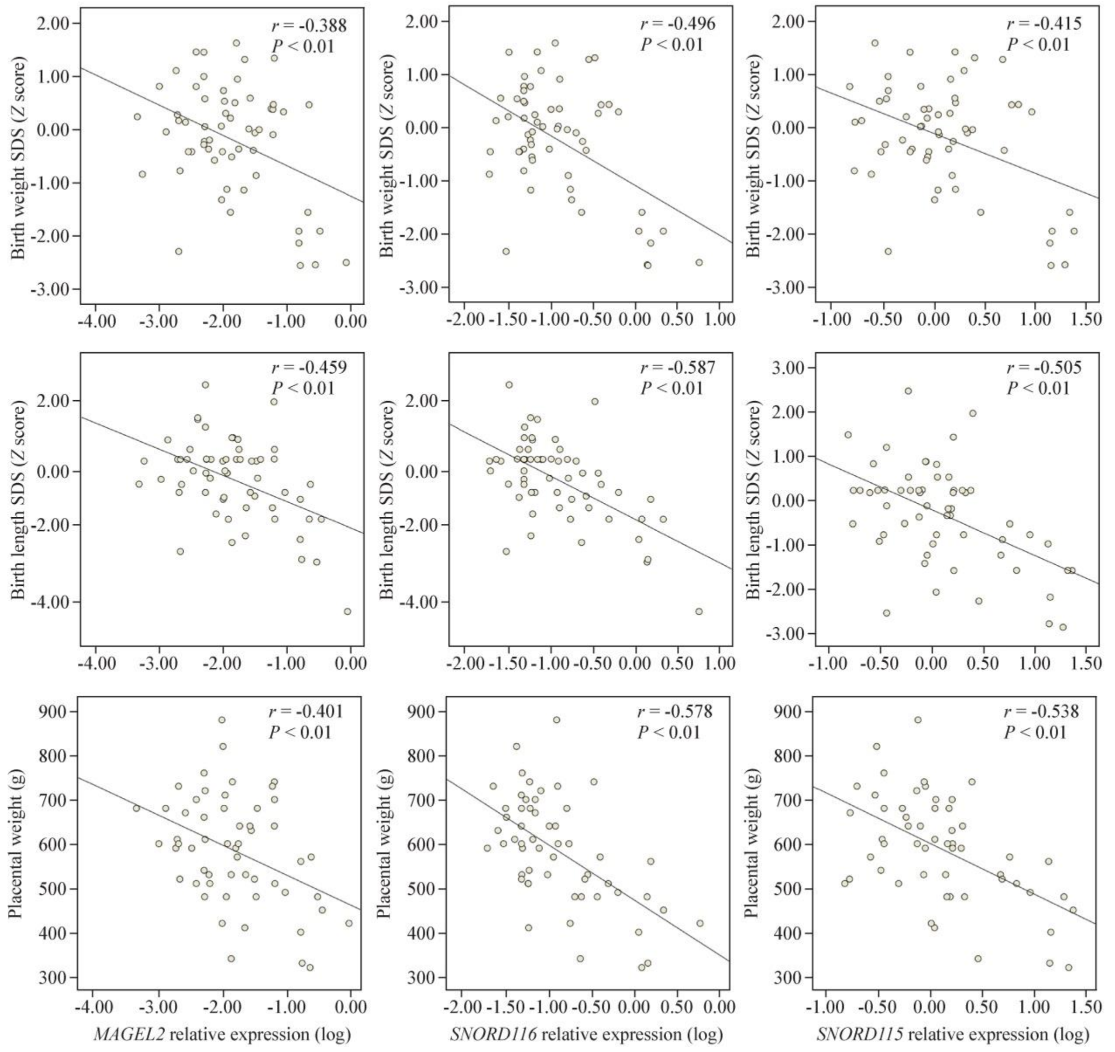

Supplementary Fig. 2 Correlations between the relative gene expression of *MAGEL2, SNORD116* and *SNORD115* in the umbilical cord and auxological variables at birth (prenatal growth) in girls. Pearson correlation coefficient (*r*) and *P* values are shown. *SDS* standard deviation score

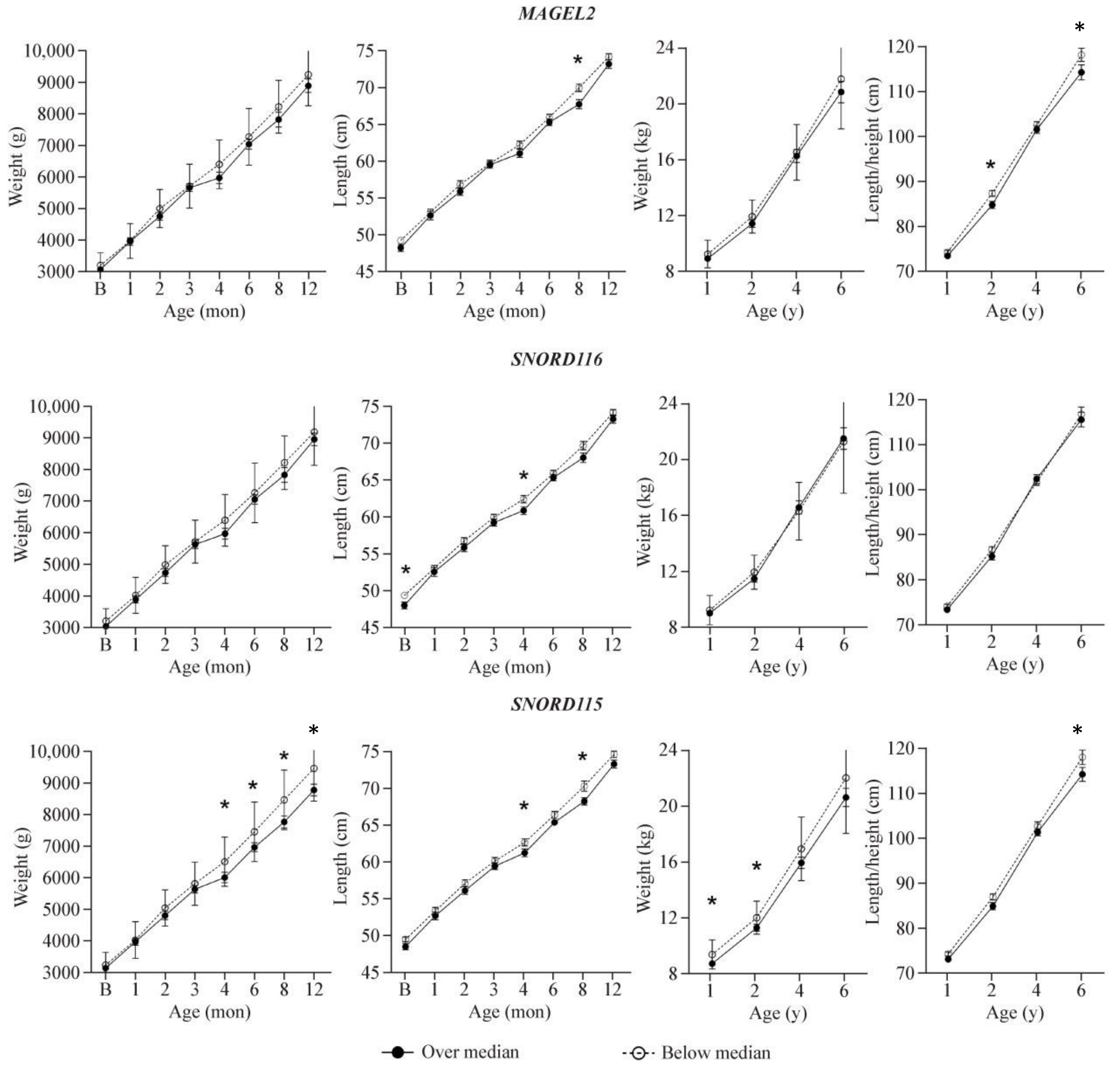

Supplementary Fig. 3 Weight and length/height curves in girls during infancy and from the first year until 6 years of age, according to a 50th centile (median) cutoff value of cord gene expression. *$P < 0.05$

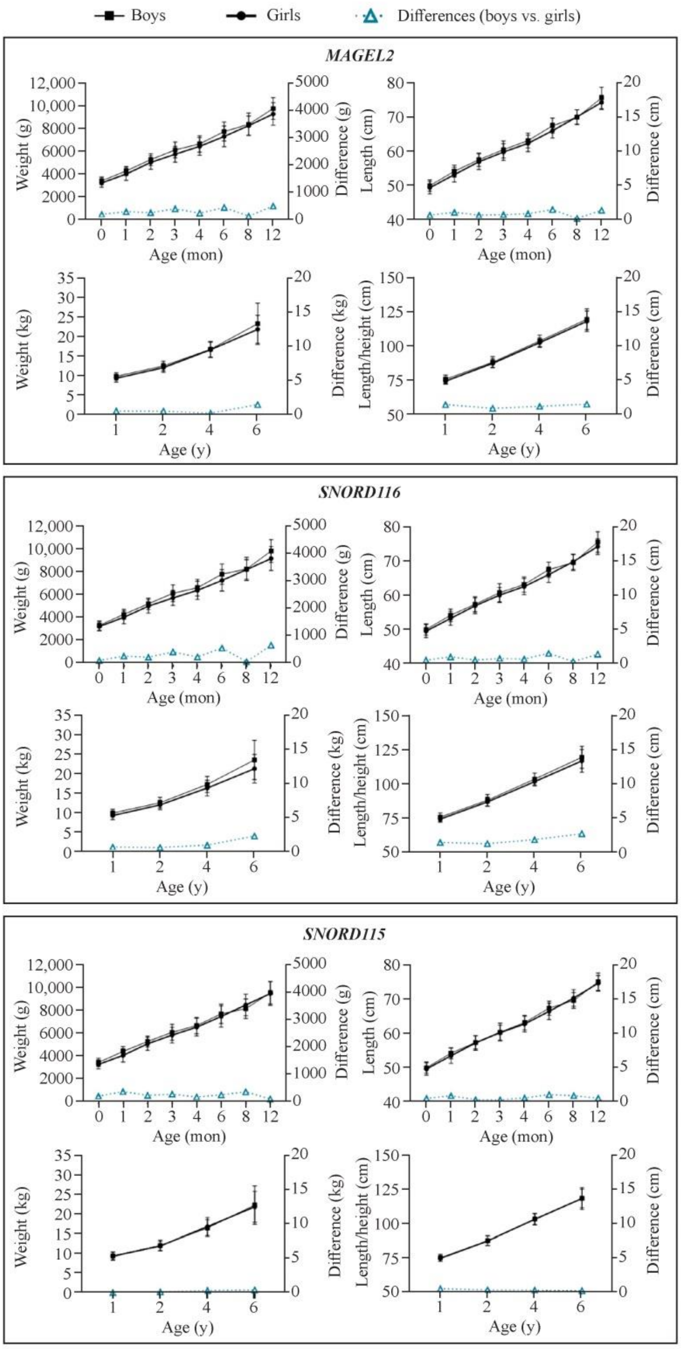

Supplementary Fig. 4 Differences between boys and girls in postnatal weight and length/height in infants with lower cord gene expression (those with expression levels below the 50th centile). $^{*}P < 0.05$
